# Improved photobio-H_2_ production regulated by artificial miRNA targeting *psbA* in green microalga *Chlamydomonas reinhardtii*

**DOI:** 10.1186/s13068-018-1030-2

**Published:** 2018-02-12

**Authors:** Hui Li, Yanmei Liu, Yuting Wang, Meirong Chen, Xiaoshan Zhuang, Chaogang Wang, Jiangxin Wang, Zhangli Hu

**Affiliations:** 1 Guangdong Technology Research Center for Marine Algal Bioengineering, Guangdong Key Laboratory of Plant Epigenetics, College of Life Sciences and Oceanography, Shenzhen University, Shenzhen, 518060 People’s Republic of China; 2 Shenzhen Key Laboratory of Marine Bioresource & Eco-environmental Science, Longhua Innovation Institute for Biotechnology, College of Life Sciences and Oceanography, Shenzhen University, Shenzhen, 518060 People’s Republic of China

**Keywords:** Biohydrogen, Microalga, *Chlamydomonas reinhardtii*, Non-coding RNA, Photosystem II

## Abstract

**Background:**

Sulfur-deprived cultivation of *Chlamydomonas reinhardtii*, referred as “two-stage culture” transferring the cells from regular algal medium to sulfur-deplete one, has been extensively studied to improve photobio-H_2_ production in this green microalga. During sulfur-deprivation treatment, the synthesis of a key component of photosystem II complex, D1 protein, was inhibited and improved photobio-H_2_ production could be established in *C. reinhardtii*. However, separation of algal cells from a regular liquid culture medium to a sulfur-deprived one is not only a discontinuous process, but also a cost- and time-consuming operation. More applicable and economic alternatives for sustained H_2_ production by *C. reinhardtii* are still highly required.

**Results:**

In the present study, a significant improvement in photobio-H_2_ production was observed in the transgenic green microalga *C. reinhardtii*, which employed a newly designed strategy based on a heat-inducible artificial miRNA (amiRNA) expression system targeting D1-encoded gene, *psbA*. A transgenic algal strain referred as “amiRNA-D1” has been successfully obtained by transforming the expression vector containing a heat-inducible promoter. After heat shock conducted in the same algal cultures, the expression of amiRNA-D1 was detected increased 15-fold accompanied with a 73% decrease of target gene *psbA*. More interestingly, this transgenic alga accumulated about 60% more H_2_ content than the wild-type strain CC-849 at the end of 7-day cultivation.

**Conclusions:**

The photobio-H_2_ production in the engineered transgenic alga was significantly improved. Without imposing any nutrient-deprived stress, this novel strategy provided a convenient and efficient way for regulation of photobio-H_2_ production in green microalga by simply “turn on” the expression of a designed amiRNA.

**Electronic supplementary material:**

The online version of this article (10.1186/s13068-018-1030-2) contains supplementary material, which is available to authorized users.

## Background

Due to the irreversible consumption of global energy reserves and the serious environmental pollution problems, hydrogen (H_2_), one of the most effective and clean fuels, is attracting tremendous attention currently [1–3]. In contrast to industrial H_2_ production mainly by steam reforming from fossil fuels, photobio-H_2_ production by green algae is of great interest because (i) it utilizes simply solar energy and water for H_2_ generation [4]; (ii) algal cells are easily cultivated by relatively cheap inorganic elements [5]; and (iii) the algal hydrogenase enzymatic conversion of H_2_ is reported possessing the highest catalytic efficiency among all known H_2_-producing organisms [6–9].

As the model organism of green algae, *Chlamydomonas reinhardtii* has been extensively used to study the phtotobio-H_2_ production in recent years [10]. In addition, a sulfur-deprived cultivation method discovered by Melis et al. solved the key barrier of phtotobio-H_2_ production by photosynthetic *C. reinhardtii* [11]. When the protein D1, one member of the photosystem II (PS II) complex, was inhibited during sulfur-deprived cultivation, it resulted in a reduced activity of PS II and thus a suppression of photosynthetic O_2_ evolution. It was also reported that a copper responsive inducible chloroplast expression system targeting Nac2 protein, which is required for the stable accumulation of the *psbD* mRNA encoding the D2 reaction center polypeptide of PS II, can be used to turn off PS II activity and thereby impose a potential for hydrogen production in *C. reinhardtii* [12]. However, the implementation of sulfur-deprivation technique needs laborious downstream process, such as cost- and time-consuming of separation of algal cells from a regular liquid culture medium to a sulfur-deprived one. Moreover, obviously this method was not a continuous process. More applicable and economic ways for the practice are still highly required [13].

microRNAs (miRNAs) are usually small RNAs 21–22 nucleotides long, but with important regulatory roles found in eukaryotes including *C. reinhardtii* [14–17]. Recently, we have identified 22 new miRNAs and their targets which were responsive to sulfur-deprivation treatment in *C. reinhardtii* [18]. In 2008, Zhao et al. [19] and Molnar et al. [20] developed a novel strategy using artificial miRNA (amiRNA) to regulate the function of given genes in *C. reinhardtii*. Since then, more amiRNA studies in *C. reinhardtii* have been conducted [21, 22], showing the promising utility of amiRNA technique on gene functional regulation in this species. In this study, since the turnover of D1 protein in PS II complex of *C. reinhardtii* has been confirmed to be correlated with the improved H_2_ yield by the sulfur-deprivation method, we proposed here a new strategy on regulation of photobio-H_2_ production by *C. reinhardtii*. With a heat-inducible amiRNA expression system targeting D1-encoded gene, *psbA* (GenBank: MF083692), a “turn on” effect corresponding to improve H_2_ production could be expected upon the induction of this amiRNA-D1 expression inside the same algal culture.

In the present study, we constructed an amiRNA-targeting *psbA* in *C. reinhardtii*. When the transgenic algae were heat-treated, difference in H_2_ production capacity was observed between the transgenic and control cells, resulting in about 60% more H_2_ accumulated at the end of the gas collection period (7 days). Without changing the culture medium, these results might suggest a convenient way for continuous H_2_ production by green microalgae.

## Results

### Selection of amiRNA candidate targeting *psbA* gene

**Fig. 1:**
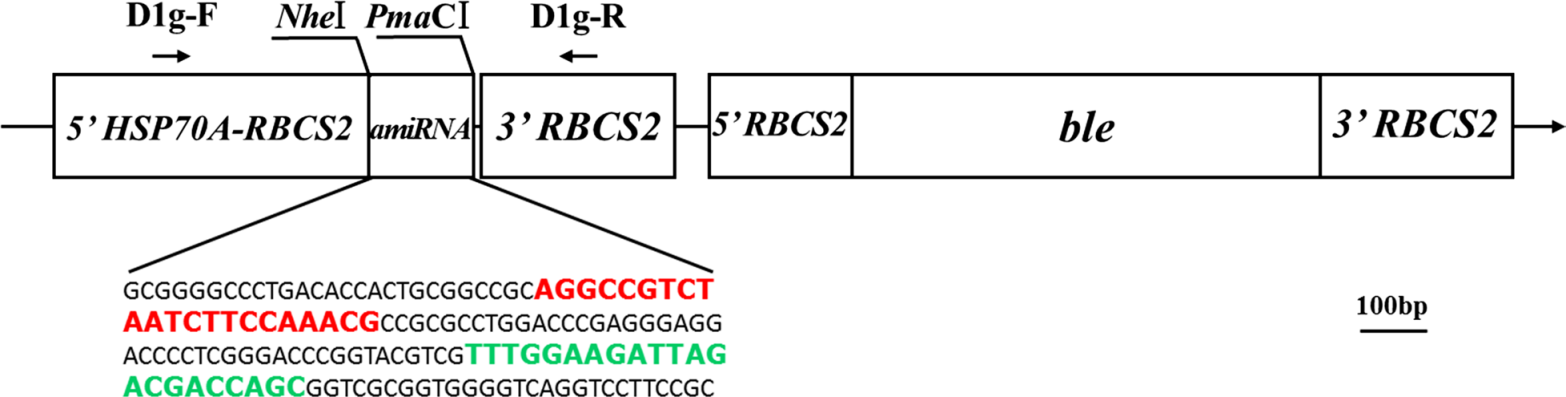
Partial scheme of expression vector pH124-amiRNA-D1

**Fig. 2:**
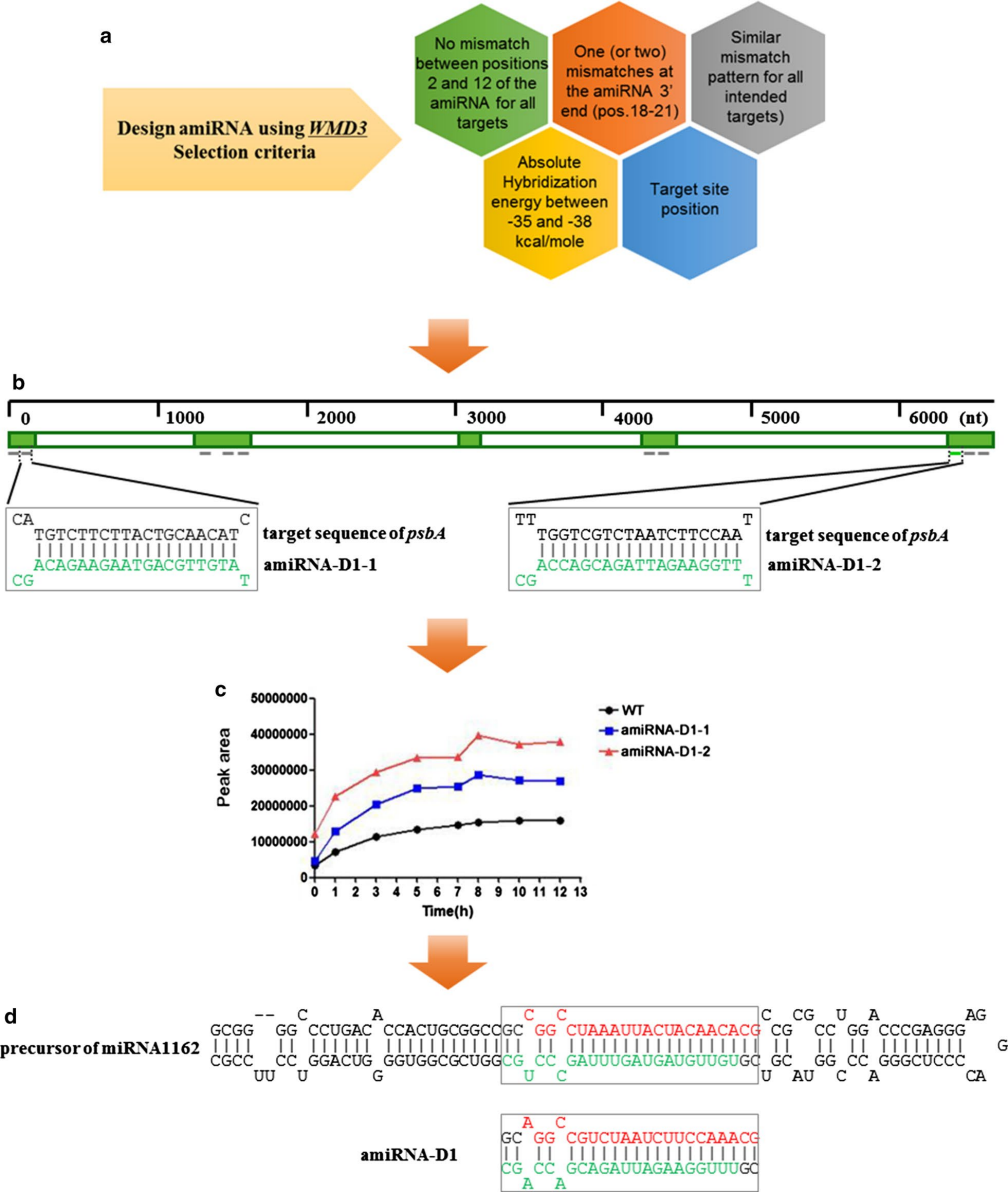
Construction of amiRNA-targeting *psbA* gene. **a** Selection criteria of WMD3 designing amiRNA. **b** Position of amiRNA-D1 target region in *psbA* gene. Solid green boxes represent *psbA* exons. Open boxes represent *psbA* introns. Short lines under the gene sketch show the designed amiRNAs, and the green line is the chosen amiRNA-D1. Target sequence of *psbA* is shown in black, and the mature miRNA sequence is shown in green. **c** Relative H_2_-producing level detected by gas chromatography (GC) indicates the first screen of *Chlamydomonas* transformants containing two best scored amiRNA candidates. **d** Loop–stem structure of miRNA1162 precursor and amiRNA-D1. The mature parts (miRNA/miRNA* duplex) are shown in the box. The mature miRNA sequence is shown in green and the miRNA* sequence is shown in red

With the help of two enzymatic digestion sites *Nhe*I and *Pma*CI, the precursor of amiRNA-D1 was inserted into the expression vector pH124 under the control of a heat-inducible promoter *HSP70A*-*RBCS2* (Fig. 1). The amiRNA-D1 sequence was designed by a web-based tool WMD3. Following selection criteria have been used to select the candidate miRNA: (i) no mismatch between positions 2 and 12 of the amiRNA for all targets; (ii) one (or two) mismatches at the amiRNA 3′ end (position 18–21); (iii) similar mismatch pattern for all intended targets; (iv) absolute hybridization energy between − 35 and − 38 kcal/mol; (v) effect of target site position on effectiveness of amiRNA (Fig. 2a, b). As a result, the sequence 5′-TATGTTGCAGTAAGAAGACAGC-3′ (amiRNA-D1-1) located at 116 and 137 nt of the target gene and the sequence 5′-TTTGGAAGATTAGACGACCAGC-3′ (amiRNA-D1-2) located at 763–784 nt of *psbA* CDS were considered as the best candidates out of 10 proposed sequences (Fig. 2b). H_2_ production detected by gas chromatography (GC) indicated that amiRNA-D1-2 should be more efficient in silencing target gene *psbA* (Fig. 2c). Accordingly, amiRNA-D1-2 (hereafter short for amiRNA-D1) was used in the following experiments. Loop–stem structure of miRNA1162 precursor and amiRNA-D1 sequences are shown in Fig. 2d.

### Identification of positive transformant expressing amiRNA-D1

**Fig. 3:**
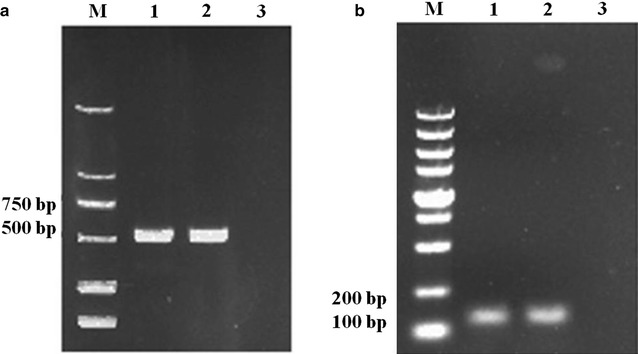
Verification of transformant using PCR and RT-PCR. **a** The PCR amplification results of amiRNA-D1 fragment in transgenic algal strain and in WT. **b** RT-PCR amplification results of amiRNA in transgenic algal strain and in WT. Lanes 1, 2: transgenic algal strain; lane 3: WT CC-849; *M* molecular marker. All the positive bands were purified and verified by sequencing. Full-length gels are presented in Additional file 1: Figure S1

About 2 weeks after algal transformation, single colonies could be observed on TAP agar plates containing antibiotics zeocin. Genomic DNA was isolated from positive transformant and PCR was performed to verify the correct integration of amiRNA-D1 on the nuclear genome of *C. reinhardtii*. We observed clearly that one fragment with expected size of 593 bp was successfully amplified, while no single PCR band could be visualized with DNA from WT (Fig. 3a). In addition, small RNAs were extracted from transgenic algae and one RT-PCR product with a size of 110 bp could be observed from Fig. 3b. The positive bands were purified and sequencing verified. These results revealed that the algal transformation was successfully performed and the amiRNA-D1 was actively transcribed.

### Effect of amiRNA transformation on algal growth

**Fig. 4:**
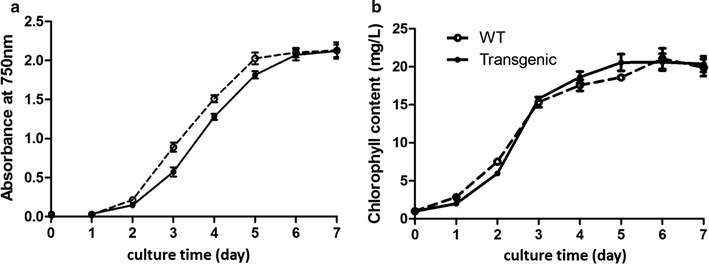
Growth curve (**a**) and chlorophyll content (**b**) of WT and transgenic algae. Data were the average of three replicates independently

The changes in growth rate and chlorophyll content of transgenic algae after algal transformation were also investigated. It was observed that the transgenic operation had no significant impact on the growth rate or chlorophyll content (Fig. 4). With a slightly slow growth rate compared to WT algae, the cell density and chlorophyll content of the transgenic alga attainted about 4–5 × 10^6^ cells/mL and 20 mg/L, respectively, at 5th day.

### Down-regulated effect of amiRNA-D1 on its target *psbA* gene

**Fig. 5:**
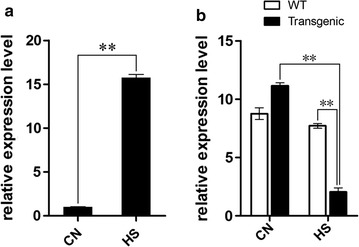
Quantitative analyses of amiRNA and its target gene at mRNA levels. Expression of amiRNA-D1 in transgenic alga (**a**) and *psbA* in WT and transgenic alga (**b**) before and after heat shock. *CN* control, *HS* heat shock. **p < 0.01

To verify the effect of heat shock treatment on expression of amiRNA-D1 and its target *psbA* gene, we used the qRT-PCR technique to detect their transcript abundance in both transgenic and WT algae. Incubation at 42 °C for 1 h has been proved to be the most efficient condition for promoting foreign gene expression driven by *HSP70A*-*RBCS2* heat-inducible promoter [23]. As shown in Fig. 5, the quantity of amiRNA-D1 transcript in transgenic algae was about 15-fold more than that in control after incubation in 42 °C for 1 h, suggesting that the inducible promoter *HSP70A*-*RBCS2* effectively promoted the expression of amiRNA-D1. Moreover, after the same heat shock treatment, the expression of *psbA* gene in transformant was about 73% lower than that in control. These results therefore confirmed the down-regulation function of constructed amiRNA-D1 against its target *psbA* gene.

### Improved H_2_-producing capacity of transgenic alga

**Fig. 6:**
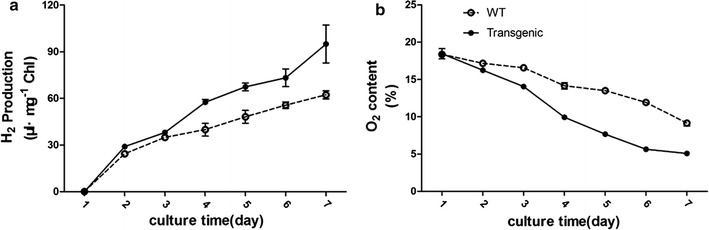
H_2_ production (**a**) and O_2_ content (**b**) of WT and transgenic alga after heat induction

To test the effect of heat-induced amiRNA-D1 expression on photobio-H_2_ production, gas chromatography was used to determine the content of gas in the headspace of WT and transgenic algal cultures. The difference in total H_2_ yield between transgenic and WT algae could be observed since 2nd day after heat induction (Table 1). The transgenic algal culture produced 48.6% (± 6.5%) more H_2_ than the WT group at 4th day and this increase in total H_2_ yield was detected until the end of the experiment. As a consequence, the transgenic algal culture reached its maximum level at 7th day, with 57.1% (± 28.6%) more H_2_ accumulated than the WT group. This promotion of total H_2_ output was also in accordance with the H_2_ production per microgram chlorophyll content (Fig. 6a). Meanwhile, the O_2_ contents in gas phase showed a more rapid O_2_ consumption in transgenic alga than the WT strain (Fig. 6b), dropping to about 4% in the mutants after 7 days while the number was more than 10% in the WT.

**Table 1:** Comparison of the total H_2_ yield (μL) in the WT strain (CC-849) and the transgenic algae (amiRNA-D1) after the heat induction

Strain	Day						
	1	2	3	4	5	6	7
WT (CC-849)	0	149.34 ± 4.38	214.08 ± 5.42	244.83 ± 25.51	295.35 ± 25.38	341.19 ± 12.69	381.99 ± 16.02
Transgenic (amiRNA-D1)	0	183.50 ± 5.20	240.71 ± 8.27	363.89 ± 11.21	426.37 ± 15.76	463.19 ± 29.28	600.25 ± 77.15

## Discussion

Sulfur-deprived cultivation of *C. reinhardtii* [11] was previously regarded as the most efficient technique to enhance photobio-H_2_ production in microalgae [24–26]. Under the sulfur-depletion condition, the amount of D1 protein was significantly reduced [26]. And thus a reduced photosystem activity was observed, resulting in a significantly prolonged H_2_ production in *C. reinhardtii* [11, 26]. In the present study, we showed for the first time a novel strategy on regulation of D1 through artificial miRNA to improve the photobio-H_2_ production in *C. reinhardtii*. Instead of making irreversible changes on D1 protein like amino acid substitutions, we chose amiRNA, a newly developed technique, to conditionally control the expression of this target gene. MiRNAs are a family of regulatory small RNAs with down-regulated function on their target genes at either transcriptional or translational level [27]. Based on the discovery of sulfur-deprivation on *C. reinhardtii* that the inhibition of D1 synthesis was correlated with the improved photobio-H_2_ output, we proposed here to achieve the similar inhibition on D1-encoded gene using amiRNA technique. Several advantages made us to express amiRNA-targeting *psbA* to regulate *C. reinhardtii* H_2_ production: (i) it has been showed that amiRNA technique in *C. reinhardtii* was successful [19–22, 28, 29]; (ii) alternatives are highly demanded for sustainable H_2_ production in *C. reinhardtii*; (iii) induction of foreign gene expression in *C. reinhardtii* was widely employed and it can be accomplished in the same culture medium [19, 20, 30–33]. The constructed vector with a heat-inducible promoter was then transformed into *C. reinhardtii* and expected properties of transformant cells were showed with a down-regulated expression of the target gene *psbA* against a corresponding up-regulated expression of amiRNA-D1. These results suggested that a successfully constructed amiRNA-D1 was functional at the post-transcriptional level in transgenic *C. reinhardtii*.

Several other studies have showed their success towards improving H_2_ production by mutating D1 protein in *C. reinhardtii* [34, 35]. Recently, Torzillo et al. showed that a *C. reinhardtii* D1 mutant strain carrying two amino acid substitutions resulted in better performance on total H_2_ output and H_2_ production duration under the sulfur-deprived condition [36]. Nevertheless, once the mutation on D1 was conducted in *C. reinhardtii*, it is an irreversible operation which means the PS II complex of these algal cells would be affected permanently. Long-term effects of the capacity of H_2_ production in the mutants such as starch accumulation could be expected, and the work of Posewitz et al. already showed that a decreased capacity in storing starch might compromise the H_2_-released duration [37]. Thus, it is obviously inappropriate for sustained algal photo-H_2_ production using D1 mutant strains. Compared with these mutants, our amiRNA-D1 obviously showed advantages since it is an inducible system.

More interestingly, the correlation between the heat induction of amiRNA-D1 expression and the improved H_2_ production capacity has been confirmed in our transgenic algae. After a period of 7-day culture after heat induction treatment, the transgenic algae produced in total about 60% more H_2_ than the WT at the end of the test (Table 1). This increase in total H_2_ output was also in accordance with the H_2_ production per microgram chlorophyll content, which was about 50% more H_2_ produced by transgenic alga at 7th day after heat induction (Fig. 6). Even though the detailed characterizations on this transgenic alga were still ongoing, the improved H_2_ production capacity after expressing amiRNA-D1 might be partially explained by a more rapid consumption of O_2_ during the test (Fig. 6). A similar effect on improvement of H_2_ production in green alga has been observed in several previously reported studies [38, 39], which used the co-cultivation of a fermentative bacterium to reduce the O_2_ content and enhance the H_2_ production in these cells. This improvement in H_2_ productivity of our transgenic algae was comparable with reported methods under sulfur-replete conditions [40, 41]; however, some factors might still account for the less significant yield of photobio-H_2_ production by this transgenic alga when compared with sulfur-deprived cells [11, 24–26] or D1 mutants [34]: (i) although a very effective knock-down effect of amiRNA-D1 on its target gene (Fig. 3), the mRNA level of *psbA* gene could not be totally silenced due to the limitation of artificial miRNA technique; (ii) pretreatment of the cultures such as sparge with argon was not conducted before the detection of the gas content, which resulted in a relatively higher O_2_ content (~ 18%) in the headspace of the cultures; (iii) the heat induction which consisted of an incubation at 42 °C for 1 h is the most common and efficient way for promoting foreign gene expression in *C. reinhardtii* [30–33]; however, this treatment before the gas detection might have negative impacts on photobio-H_2_ production performance; (iv) the mechanisms of action of non-coding RNA in *C. reinhardtii* still need further clarification based on the limited reports [18, 42]. Even though our study provided a potential for hydrogen production using amiRNA targeting a PSII protein, the amounts of H_2_ production under current experimental conditions were still relatively low. The optimization of treatment conditions and gas detections are underway in our laboratory.

In a summary, our study added evidence to the possibility of employing designed amiRNA to enhance the photobio-H_2_ production in green microalgae, without the trouble of changing the culture medium.

## Methods

### Algal strains, culture conditions and gas detection

A cell-wall-deficient *C. reinhardtii* strain, CC-849, was obtained from the *Chlamydomonas* Genetic Center of Duke University (Duke University, Durham, NC, USA) and served as the wild-type (WT) strain. Cells were cultured in TAP (Tris–acetate–phosphate) medium at 25 °C and under continuous cool-white fluorescent lamps (≈ 200 μmol photons/m^2^/s). Antibiotic was only used for positive transgenic colonies. Once the positive colonies were verified, cells were cultivated in normal medium without antibiotics. For conducting heat shock induction, 400 mL cells (WT and transgenic, cell density of ≈ 1 × 10^7^ cells/mL) were incubated in a water bath at 42 °C for 1 h. After the treatment, algal cells were subjected for gas detection and quantitative real-time PCR analysis. Algal cells for H_2_ detection and physiological analysis were sampled from different bottles, since the bottles for H_2_ detection were sealed until the end of the experiment.

Cells of the WT and transgenic algae (400 mL) at mid-logarithmic phase were transferred into glass bottles with a total volume of 500 mL, and then sealed with the gas-tight septum. The cultures were incubated in dark for 24 h before detecting the gas content in the headspaces, and then the cells were cultivated under continuous light. Three bottles of transgenic strain and three bottles of CC-849 were set for H_2_ detection after heat induction. All these six bottles were sampled at each time point, and then was placed back to normal growth condition under continuous white light at 25 °C. A gas chromatograph with a thermal conductivity detector was used to determine the concentration of H_2_ and O_2_ (Agilent 7890A; Agilent Technologies Inc., USA). 1 mL of gas in the headspace of the cultures was sampled with a gas-tight syringe. Gas samples were next separated by a molecular sieve column (type 5 Å; 2 m × 1/8 mm), and argon was used as the carrier gas.

### Algal growth and chlorophyll measurement

Cells in the liquid TAP media (cell density of ≈ 1 × 10^7^ cells/mL) were inoculated into the new culture flasks with triplicates. The growth and chlorophyll content of these new cultures were recorded each day during a 7-day monitoring. The algal cell growth was monitored by spectrophotometer (UV-1800, MAPADA Instruments, Shanghai, China) at 750 nm. For the chlorophyll measurement of algal cells, Spreitzer’s method [43] was applied after a step of 95% ethanol extraction.

### amiRNA constructs and algal transformation

The expression vector pH124 containing the ampicillin- and zeocin-resistant genes and a strong heat-inducible promoter (*HSP70A*-*RBCS2)* was constructed and maintained in the College of Life Sciences, Shenzhen University. Pre-amiRNA-D1 was constructed into the pH124 based on the backbone of the precursor of miRNA1162 as previously described [19], mainly for its highly expressed property in *C. reinhardtii*. The vector pH124 system will randomly integrate the promoters, amiRNA and the antibiotics marker together into the nuclear genome [20]. Enzymatic digestion sites of *Nhe*I (GCTAGC) and *Pma*CI (CACGTG) were added at 5′- and 3′-end of pre-amiRNA-D1, respectively, and the mature part of the precursor of miRNA1162 was replaced by that of amiRNA-D1 (Fig. 1).

To select the most appropriate sequence targeting to *psbA* gene, a web-based tool for amiRNA design was used to develop our mature amiRNA-D1 (WMD3, http://wmd3.weigelworld.org/cgi-bin/webapp.cgi). amiRNA-D1 (5′-TATGTTGCAGTAAGAAGACAG-3′) is complementary to nucleotides 117–137 bp of the coding region of *psbA*, while the amiRNA-D1* sequence (5′-CTGTCTTCTTACTGCAACATA-3′) is used to maintain the structure of the amiRNA duplex. The constructed pre-amiRNA-D1 (155 bp) was commercially synthesized in vitro and was cloned in plasmid pUC57 (Sangon Biotech Co., Ltd, Shanghai, China). The final construction of pH124-amiRNA-D1 was completed by inserting pre-amiRNA-D1 into an empty pH124 vector with the help of two previously designed recognition sites *Nhe*I and *Pma*CI (Fig. 2). Algal transformation with pH124-amiRNA-D1 was conducted using the glass-bead method as previously described [32].

### DNA-PCR analysis on transgenic alga

Genomic DNA was extracted from both WT and transgenic algae using the DNeasy kit (Takara, Japan). PCR was performed according to the standard protocols [44] to verify the presence of amiRNA-D1 on transgenic alga. PCR was performed using the primer pair D1g-F (5′-TGACCTCCACTTTCAGCGACA-3′) and D1g-R (5′-ACTTGAGAGCAGTATCTTCCATCCA-3′), which resulted in an amplicon of about 600 bp. PCR conditions were incubated at 94 °C for 5 min, followed by 25 cycles of 94 °C for 30 s, 55 °C for 30 s, and 72 °C for 30 s, plus a final extension for 7 min. All the amplified products were purified and verified by sequencing analyses (Sangon Biotech., Shanghai, China).

### RNA extraction and RT-PCR

WT and transgenic algae were harvested and were incubated at 42 °C for 1 h before extraction of total RNA. RT-PCR analysis was next performed to verify the expression of the target gene *psbA* and amiRNA. Total RNA from both WT and transgenic alga was isolated, respectively, using the TRIZOL reagent (Invitrogen, Life Technologies, Carlsbad, CA). High molecular weight RNA and low molecular weight RNA were separated as previously reported [18]. For the first group RNA, classic reverse-transcriptase M-MLV and random primer (Takara, Japan) were used to accomplish the reverse-transcription according to the manufacturer’s protocol. For the second group mainly small RNAs, polyadenylation and transcription were performed according to *S*-Poly(T) method [45]. Briefly, reverse-transcription was initialed by a step of polyadenylation at 37 °C for 60 min in a 50-μL reaction mixture with 1.5 μg of total RNA, 1 mM ATP, 2.5 mM MgCl_2_, and 4 U poly(A) polymerase (Takara, Japan). Phenol/chloroform extraction and ethanol precipitation were next used to recover the Poly(A)-tailed sRNA. After treated with RNase-free DnaseI (Takara, Japan), the sRNAs were reversely transcribed using poly (T) adapter (5′-GTGCAGGGTCCGAGGTCAGAGCCACCTGGGCAATTTTTTTTTTTCTGTCT-3′). The final RT-PCR was realized using a universal reverse primer (5′-CAGTGCAGGGTCCGAGGT-3′) and the specific forward primer (5′-TGTCGGTATGTTGCAGTAAGA-3′), which resulted in an amplicon of about 110 bp. The amplified positive products were verified by sequencing analyses (Sangon Biotech., Shanghai, China).

### Quantitative real-time PCR (qRT-PCR)

To quantitatively detect the change of amiRNA-D1 and *psbA* expression in both WT and transgenic alga, qRT-PCR was performed with Applied Biosystems 7300 real-time PCR System (Framingham, MA, USA), and the primers used are listed in Table 2. RNA extraction from the heat-shocked WT and transgenic algae was as previously described for “RT-PCR analysis”, while the RNA extracted from the untreated WT and transgenic algae was used as the control. The standard protocol was applied to *psbA* expression detection using SYBR Premix Ex Taq™ II (Takara, Japan) according to the manufacturer’s instruction, while the expression of amiRNA-D1 was detected as previously described [45]. Briefly, using SYBR Green Real-time PCR Master Mix (Toyobo, Osaka, Japan), each reaction containing 1 μL of diluted cDNA (about 100 pg of RNA, previously reverse-transcribed by *S*-Poly(T) method), 10 μL of 2× SYBR green reaction mix, and 5 pmol of the forward and reverse primers were added to make a final volume of 20 μL. PCR conditions were as follows: one step of 95 °C for 30 s, followed by 40 cycles of 95 °C for 5 s and 60 °C for 30 s. Analysis of the melting curve of amplicons was used to test the specificity of the primers. The actin gene and U4 snoRNA were used as a reference gene in the qRT-PCR detection of *psbA* and amiRNA-D1, respectively. The data with an R^2^ above 0.998 were analyzed using the 2^−ΔΔCt^ program [46].

**Table 2:** Primers used for amiRNA-D1 and *psbA* gene expression detections by qRT-PCR

Name	Primer	Sequence (5′ → 3′)
*psbA* quantification		
D1-F	Forward primer	CGGTAATCGGTATTTGGT
D2-R	Reverse primer	GCACGGTTGATGATGTCT
Actin-F	Forward primer	ACCCCGTGCTGCTGACTG
Actin-R	Reverse primer	ACGTTGAAGGTCTCGAACA
amiRNA-D1 quantification		
miRNA-D1-F	Forward primer	TGTCGGTATGTTGCAGTAAGA
RT-amiRNA-D1	Primer for reverse-transcription	GTGCAGGGTCCGAGGTCAGAGCCACCTGGGCAATTTTTTTTTTTCTGTCT
U4-F	Forward primer	CGGCGCAAAAGGCCCGACAGAAAT
RT-U4	Primer for reverse-transcription	GTGCAGGGTCCGAGGTCAGAGCCACCTGGGCAATTTTTTTTTTTATTTCTC
Universal R	Universal reverse primer	CAGTGCAGGGTCCGAGGT

### Statistical analysis

All experiments were repeated at least three times independently, and data were recorded as the mean with standard deviation (SD). Statistical analyses were performed using the Student’s *t* test and Pearson correlation analysis (SPSS13.0). For all of the data analysis, a *p* value < 0.05 was considered statistically significant.

## Additional file

**Additional file 1: Figure S1.** Full-length gels of Fig. 3.

## References

[1] Bockris J (2002). The origin of ideas on a hydrogen economy and its solution to the decay of the environments. Int J Hydrogen Energy.

[2] Dunn S (2002). Hydrogen futures: toward a sustainable energy system. Int J Hydrogen Energy.

[3] Lakaniemi AM, Hulatt CJ, Thomas DN, Tuovinen OH, Puhakka JA (2011). Biogenic hydrogen and methane production from *Chlorella vulgaris* and *Dunaliella tertiolecta* biomass. Biotechnol Biofuels.

[4] Yang S, Guarnieri MT, Smolinski S, Ghirardi M, Pienkos PT (2013). *De novo* transcriptomic analysis of hydrogen production in the green alga *Chlamydomonas moewusii* through RNA-Seq. Biotechnol Biofuels.

[5] Turner JA (2004). Sustainable hydrogen production. Science.

[6] Forestier M, King P, Zhang L, Posewitz M, Schwarzer S, Happe T (2003). Expression of two [Fe]-hydrogenases in *Chlamydomonas reinhardtii* under anaerobic conditions. Eur J Biochem.

[7] Happe T, Mosler B, Naber JD (1994). Induction, localization and metal content of hydrogenase in the green alga *Chlamydomonas reinhardtii*. Eur J Biochem.

[8] Adams MW (1990). The structure and mechanism of iron hydrogenases. Biochim Biophys Acta.

[9] Happe T, Naber JD (1993). Isolation, characterization, and N-terminal amino acid sequence of hydrogen from the green algae *Chlamydomonas reinahrdtii*. Eur J Biochem.

[10] Esquivel MG, Amaro HM, Pinto TS, Fevereiro PS, Malcata FX (2011). Efficient H_2_ production via *Chlamydomonas reinhardtii*. Trends Biotechnol.

[11] Melis A, Zhang L, Forestier M, Ghirardi ML, Seibert M (2000). Sustained photobiological hydrogen gas production upon reversible inactivation of oxygen evolution in the green alga *Chlamydomonas reinhardtii*. Plant Physiol.

[12] Surzycki R, Cournac L, Peltier G, Rochaix JD (2007). Potential for hydrogen production with inducible chloroplast gene expression in *Chlamydomonas*. PNAS.

[13] Hwang JH, Kim HC, Choi JA, Abou-Shanab RA, Dempsey BA, Regan JM (2014). Photoautotrophic hydrogen production by eukaryotic microalgae under aerobic conditions. Nat Commun.

[14] Zhao T, Li G, Mi S, Li S, Hannon GJ, Wang XJ (2007). A complex system of small RNAs in the unicellular green alga *Chlamydomonas reinhardtii*. Genes Dev.

[15] Molnar A, Schwach F, Studholme DJ, Thuenemann EC, Baulcombe DC (2007). miRNAs control gene expression in the single-cell alga *Chlamydomonas reinhardtii*. Nature.

[16] Hajieghrari B, Farrokhi N, Goliaei B, Kavousi K (2016). Identification and characterization of novel miRNAs in *Chlamydomonas reinhardtii* by computational methods. MicroRNA.

[17] Voshall A, Kim EJ, Ma X, Yamasaki T, Moriyama EN, Cerutti H (2017). miRNAs in the alga *Chlamydomonas reinhardtii* are not phylogenetically conserved and play a limited role in responses to nutrient deprivation. Sci Rep.

[18] Shu LF, Hu ZL (2012). Characterization and differential expression of microRNAs elicited by sulfur deprivation in *Chlamydomonas reinhardtii*. BMC Genomics.

[19] Zhao T, Wang W, Bai X, Qi Y (2009). Gene silencing by artificial microRNAs in *Chlamydomonas*. Plant J..

[20] Molnar A, Bassett A, Thuenemann E, Schwach F, Karkare S, Ossowski S (2009). Highly specific gene silencing by artificial microRNAs in the unicellular alga *Chlamydomonas reinhardtii*. Plant J..

[21] Burgess SJ, Tredwell G, Molnàr A, Bundy JG, Nixon PJ (2012). Artificial microRNA-mediated knockdown of pyruvate formate lyase (PFL1) provides evidence for an active 3-hydroxybutyrate production pathway in the green alga *Chlamydomonas reinhardtii*. J Biotechnol.

[22] Ferrante P, Ballottari M, Bonente G, Giuliano G, Bassi R (2012). LHCBM1 and LHCBM2/7 polypeptides, components of major LHCII complex, have distinct functional roles in photosynthetic antenna system of *Chlamydomonas reinhardtii*. J Biol Chem.

[23] Schroda M, Blöcker D, Beck CF (2000). The HSP70A promoter as a tool for the improved expression of transgenes in *Chlamydomonas*. Plant J..

[24] Hemschemeier A, Fouchard S, Cournac L, Peltier G, Happe T (2008). Hydrogen production by *Chlamydomonas reinhardtii*: an elaborate interplay of electron sources and sinks. Planta.

[25] Melis A, Happe T (2004). Trails of green alga H_2_-production research-from Hans Gaffron to new frontiers. Photosyn Res..

[26] Zhang L, Happe T, Melis A (2002). Biochemical and morphological characterization of sulfur-deprived and H_2_-producing *Chlamydomonas reinhardtii* (green alga). Planta.

[27] Carrington JC, Ambros V (2003). Role of microRNAs in plant and animal development. Science.

[28] Wang C, Chen X, Li H, Wang J, Hu Z (2017). Artificial miRNA inhibition of phosphoenolpyruvate carboxylase increases fatty acid production in a green microalga *Chlamydomonas reinhardtii*. Biotechnol Biofuels.

[29] Wang Y, Jiang X, Hu C, Sun T, Zeng Z, Cai X (2017). Optogenetic regulation of artificial microRNA improves H_2_ production in green alga *Chlamydomonas reinhardtii*. Biotechnol Biofuels.

[30] Wang C, Hu Z, Lei A, Jin B (2010). Biosynthesis of poly-3-hydroxybutyrate (phb) in the transgenic green alga *Chlamydomonas reinhardtii*. J Phycol.

[31] Hou Q, Qiu S, Liu Q, Tian J, Hu Z, Ni J (2013). Selenoprotein-transgenic *Chlamydomonas reinhardtii*. Nutrients..

[32] Li H, Zhang L, Shu L, Zhuang X, Liu Y, Chen J (2015). Sustainable photosynthetic H_2_-production mediated by artificial miRNA silencing of OEE2 gene in green alga *Chlamydomonas reinhardtii*. Int J Hydrogen Energy.

[33] Mu F, Li H, Hu Z (2012). Expression of tandem repeat Cecropin B in *Chlamydomonas reinhardtii* and its antibacterial effect. Prog Biochem Biophys.

[34] Scoma A, Krawietz D, Faraloni C, Giannelli L, Happe T, Torzillo G (2012). Sustained H_2_ production in a *Chlamydomonas reinhardtii* D1 protein mutant. J Biotechnol.

[35] Makarova VV, Kosourov S, Krendeleva TE, Semin BK, Kukarskikh GP, Rubin AB (2007). Photoproduction of hydrogen by sulfur-deprived *C. reinhardtii* mutants with impaired photosystem II photochemical activity. Photosyn Res.

[36] Torzillo G, Scoma A, Faraloni C, Ena A, Johaninngmeier U (2009). Increased hydrogen photoproduction by means of a sulfur-deprived *Chlamydomonas reinhardtii* D1 protein mutant. Int J Hydrogen Energy.

[37] Posewitz MC, Smolinski SL, Kanakagiri S, Melis A, Seibert M, Ghirardi ML (2004). Hydrogen photoproduction is attenuated by disruption of an isoamylase gene in *Chlamydomonas reinhardtii*. Plant Cell..

[38] Kawaguchi H, Hashimoto K, Hirata K, Miyamoto K (2001). H_2_ production from algal biomass by a mixed culture of *Rhodobium marinum* A-501 and *Lactobacillus amylovorus*. J Biosci Bioeng.

[39] Xu L, Cheng X, Wu S, Wang Q (2017). Co-cultivation of *Chlamydomonas reinhardtii* with *Azotobacter chroococcum* improved H_2_ production. Biotechnol Lett.

[40] Wu S, Yan G, Xu L, Wang Q, Liu X (2010). Improvement of hydrogen production with expression of *lba* gene in chloroplast of *Chlamydomonas reinhardtii*. Int J Hydrogen Energy.

[41] Wu S, Xu L, Huang R, Wang Q (2011). Improved biohydrogen production with an expression of codon-optimized *hemH* and *lba* genes in the chloroplast of *Chlamydomonas reinhardtii*. Bioresour Technol.

[42] Li H, Wang Y, Chen M, Xiao P, Hu C, Zeng Z (2016). Genome-wide long non-coding RNA screening, identification and characterization in a model microorganism *Chlamydomonas reinhardtii*. Sci Rep.

[43] Hoober JK (1989). The Chlamydomonas sourcebook: a comprehensive guide to biology and laboratory use.

[44] Sambrook J, Maniatis TE, Fritsch EF (1989). Molecular cloning: a laboratory manual.

[45] Kang K, Zhang X, Liu H, Wang Z, Zhong J, Huang Z (2012). A novel real-time PCR assay of microRNAs using S-Poly(T), a specific oligo(dT) reverse transcription primer with excellent sensitivity and specificity. PLoS ONE.

[46] Livak KJ, Schmittgen TD (2001). Analysis of relative gene expression data using real-time quantitative PCR and the 2^−ΔΔCt^ method. Methods.

