# Supplementary figures and images for: Improved photobio-H2 production regulated by artificial miRNA targeting psbA in green microalga Chlamydomonas reinhardtii
Source: Biotechnol Biofuels. 2018 Feb 12;11:36. doi: 10.1186/s13068-018-1030-2 (PMC5808451; doi:10.1186/s13068-018-1030-2)

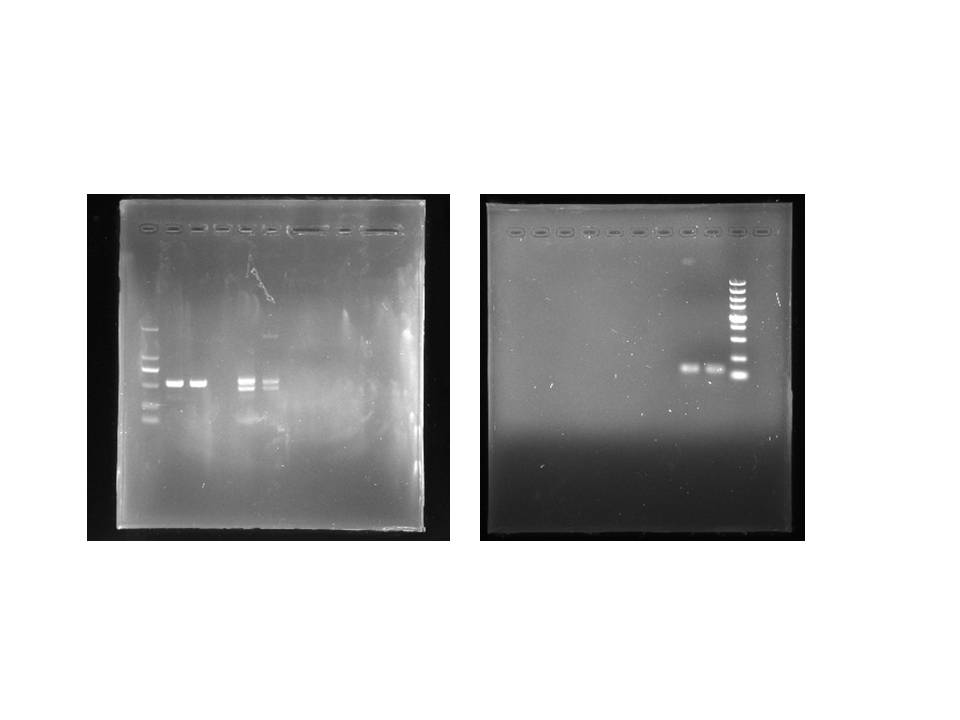

Supplement: Supplementary file 1 — Additional file 1: Figure S1. Full-length gels of Fig. 3. [file 13068_2018_1030_MOESM1_ESM.jpg]
